# Vitreous phenotyping distinguishes Wagner syndrome from ocular-only Stickler syndrome: a case series

**DOI:** 10.1038/s41433-026-04667-y

**Published:** 2026-06-29

**Authors:** David C. S. Wong, Olivia S. Knutson, Thomas R. W. Nixon, Annie M. McNinch, Howard Martin, Allan J. Richards, Martin P. Snead

**Affiliations:** 1 Vitreoretinal Research Group, John van Geest Centre for Brain Repair, Forvie Site, University of Cambridge, Cambridge, UK; 2 Vitreoretinal Service, Cambridge University Hospitals NHS Foundation Trust, Cambridge, UK; 3 NHS England Stickler Syndrome Highly Specialised Service, Cambridge University Hospitals NHS Foundation Trust, Cambridge, UK

**Keywords:** Retinal diseases, Disease genetics

Wagner syndrome and ocular-only Stickler syndrome are distinct hereditary vitreoretinopathies. They are frequently confused because they share features of congenital myopia, abnormal vitreous architecture, and retinal detachment without systemic manifestations. Indeed, the confusing and misleading diagnosis of ‘Wagner-Stickler syndrome’ still exists in some widely used databases in current use. The original Wagner syndrome pedigree did not suffer from retinal detachment [1], but such cases have since been reported [2]. Wagner syndrome is caused by splice variants in the versican (*VCAN*) gene, which encodes a major proteoglycan component in the vitreous [2]. Ocular-only Stickler syndrome typically arises from alternatively spliced variants of Type II collagen (*COL2A1*) and exhibits a characteristic membranous vitreous anomaly [3]. Accurate distinction is critical for genetic counselling and surgical planning.

We describe a multigenerational family carrying a 30-year clinical diagnosis of ocular-only Stickler syndrome, reclassified to Wagner syndrome following vitreous phenotyping and subsequent confirmatory molecular testing. Written consent was obtained. Clinical examination was supplemented by ultra-widefield retinal photography (Optos) and spectral-domain optical coherence tomography (OCT, Zeiss). Genetic analysis used the R45.1 Stickler syndrome panel from the National Genomic Test Laboratory (East Genomic Laboratory Hub, Cambridge, UK).

Four affected members across two generations were assessed (Fig. 1). All had congenital high myopia. Three had suffered retinal detachments: the proband (II-2, childhood, left eye, Fig. 2A), the father (I-1, 1993 bilateral, Fig. 2B) and the younger sister (II-4, 2023, right eye, three procedures including pars plana vitrectomy, cryotherapy and silicone oil tamponade, Fig. 2C). One sister was unaffected by retinal detachment but had early onset cataract (II-3, Fig. 2D). None had palatal, auditory or musculoskeletal involvement, and none exhibited the type 1 membranous vitreous anomaly. All four demonstrated thickened, retracted posterior hyaloid membranes and partial foveal posterior hyaloid separation typical of Wagner syndrome (Fig. 2) [2]. Confirmatory genetic testing for all four individuals identified a heterozygous *VCAN* splice-acceptor variant (NM_004385.5:c.4004-1G>A).

**Fig. 1:**
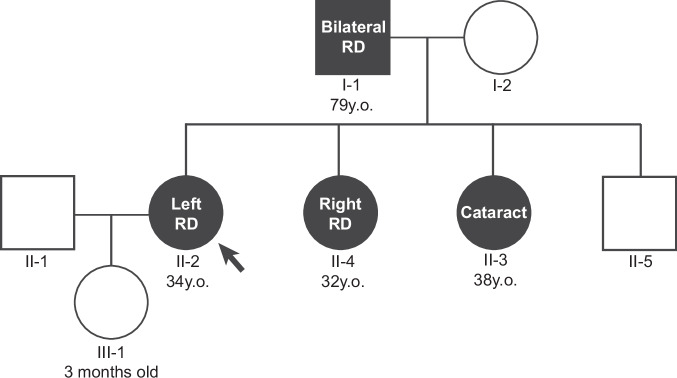
Family pedigree. All affected individuals were found to have the *VCAN* c.4004-1G>A splice-acceptor variant. RD retinal detachment.

**Fig. 2:**
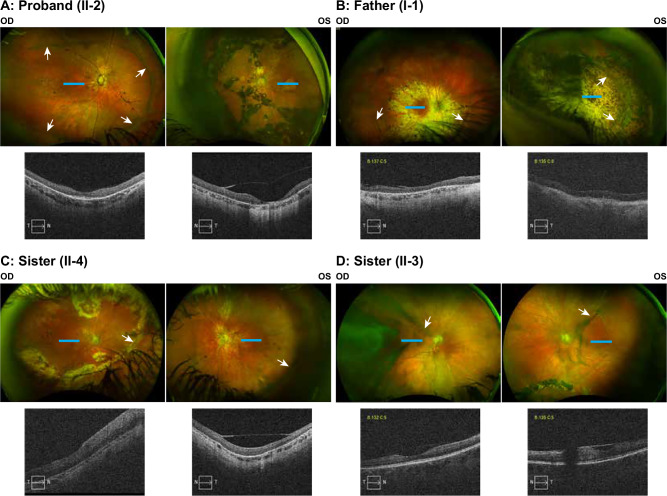
Optos widefield photography and OCT imaging. **A** *Proband (II-2)* A classic circumferential avascular vitreous veil in the right eye is present (white arrows). The left eye shows severe chorioretinal atrophy and macular scar. Corresponding macular OCT scans (cross-sections at the level of the blue lines in the photographs) below show myopic fundi and a characteristic partial posterior vitreous detachment over the left fovea without perifoveal separation, and underlying scarring. **B** *Father (I-1)* Bilateral severe chorioretinal atrophy and pale optic discs are evident, associated with severe atrophy on the macular OCT scans below. Of note, avascular circumferential vitreous veils are visible. **C** *Younger sister (II-4)* Photographs show scarring in the right eye from multiple retinal detachment repair surgeries. The left eye shows vitreous veil on photography and partial foveal posterior vitreous detachment on macular OCT. **D** *Older sister (II-3)* Bilateral chorioretinal atrophy and vitreous veils with adhesion to optic discs were found along with a right cortical cataract. Corresponding macular OCTs show partial posterior vitreous detachments over the foveas in both eyes.

There are two main implications from this case series. First, vitreous phenotyping reliably distinguishes Wagner syndrome from ocular-only Stickler syndrome and can pre-empt molecular testing. Second, the high frequency of retinal detachment in this pedigree, consistent with other recent reports [4], challenges the historical view of Wagner syndrome as a non-detaching vitreoretinopathy and supports inclusion of *VCAN* in Stickler syndrome diagnostic panels.

A reasonable question is whether the high retinal detachment burden in some families with Wagner syndrome justifies extending the Cambridge prophylactic 360° cryoretinopexy protocol used in type 1 Stickler syndrome [5]. The rationale of this treatment is to prevent the development of giant retinal tears, which are the dominant mechanism of retinal detachment in Stickler syndrome. Retinal detachments in Wagner syndrome, by contrast, arise predominantly from traction driven by abnormal posterior hyaloid adhesion, not giant retinal tears [2, 4]. Prophylactic cryoretinopexy would therefore not prevent this pathological process.

Accurate phenotypic diagnosis directly informs surgical planning, and comprehensive molecular diagnostics guide genetic counselling. As the use of gene panel testing expands, careful clinical vitreous phenotyping continues to remain essential to recognising and appropriately managing *VCAN*-associated retinal detachment.

## Data Availability

All data generated or analysed during this study are included in this published article.
